# High-Resolution Screening for Marine Prokaryotes and Eukaryotes With Selective Preference for Polyethylene and Polyethylene Terephthalate Surfaces

**DOI:** 10.3389/fmicb.2022.845144

**Published:** 2022-04-12

**Authors:** Katherine S. Marsay, Yuri Koucherov, Keren Davidov, Evgenia Iankelevich-Kounio, Sheli Itzahri, Mali Salmon-Divon, Matan Oren

**Affiliations:** ^1^Department of Molecular Biology, Ariel University, Ariel, Israel; ^2^The Adelson School of Medicine, Ariel University, Ariel, Israel

**Keywords:** DNA metabarcoding, nanopore, MinION, plastic microbiome, marine fungi, differentially abundant species, plastisphere

## Abstract

Marine plastic debris serve as substrates for the colonization of a variety of prokaryote and eukaryote organisms. Of particular interest are the microorganisms that have adapted to thrive on plastic as they may contain genes, enzymes or pathways involved in the adhesion or metabolism of plastics. We implemented DNA metabarcoding with nanopore MinION sequencing to compare the 1-month-old biomes of hydrolyzable (polyethylene terephthalate) and non-hydrolyzable (polyethylene) plastics surfaces vs. those of glass and the surrounding water in a Mediterranean Sea marina. We sequenced longer 16S rRNA, 18S rRNA, and ITS barcode loci for a more comprehensive taxonomic profiling of the bacterial, protist, and fungal communities, respectively. Long read sequencing enabled high-resolution mapping to genera and species. Using previously established methods we performed differential abundance screening and identified 30 bacteria and five eukaryotic species, that were differentially abundant on plastic compared to glass. This approach will allow future studies to characterize the plastisphere communities and to screen for microorganisms with a plastic-metabolism potential.

## Introduction

Marine plastic debris is a growing global pollution concern, as it jeopardizes aquatic life through entanglement, ingestion, or introduction of toxic chemicals [reviewed in Amaral-Zettler et al. (2020)]. Most plastic polymers persist for a long time in the oceans. As such, they serve as substrates for the colonization of a variety of marine organisms and the establishment of complex microorganism communities (Jacquin et al., 2019). This new human-made ecosystem is referred to as the plastisphere and includes a distinct biota from that of its surrounding waters (e.g., Zettler et al., 2013; Bryant et al., 2016; De Tender et al., 2017). Some marine bacteria inhabiting marine plastic debris have capabilities to degrade plastic polymers [reviewed in Roager and Sonnenschein (2019)] and few were shown to utilize plastics as their carbon food source (Sudhakar et al., 2008; Harshvardhan and Jha, 2013; Auta et al., 2017).

A suitable approach to investigate the assembly of the plastisphere communities is incubation experiments, where surfaces made of known materials are suspended in water for predefined time periods, either at sea (e.g., Eich et al., 2015; Oberbeckmann et al., 2016; Pollet et al., 2018; Pinto et al., 2019; Dudek et al., 2020) or in marine microcosms (e.g., Ogonowski et al., 2018; Kirstein et al., 2019). To identify organisms that preferentially colonize plastic debris, as opposed to general surface colonizers, reference substrates such as glass are often used (Oberbeckmann et al., 2016; Kirstein et al., 2018, 2019; Ogonowski et al., 2018; Pinto et al., 2019). The identification of organisms is usually done with DNA metabarcoding based on relevant genetic loci. Most plastisphere microbiome studies have used the hypervariable V3–V5 region of the 16S rRNA gene for metabarcoding of bacteria and the V4 or V9 regions of the 18S rRNA gene to identify eukaryotes [reviewed in Jacquin et al. (2019) and Latva et al. (2021)]. However, in most cases, the mapping of these relatively short (less than 0.5 Kbp) sequences to the databases could not resolve lower taxonomic levels and could not identify species. Additionally, the 18S rRNA gene barcodes and primers are limited in their coverage and do not cover certain phylogenetic groups such as fungi (Leray and Knowlton, 2016; Davidov et al., 2020). To date, only a handful of studies have assessed the fungal component of the plastisphere (De Tender et al., 2017; Kettner et al., 2017; Amend et al., 2019; Davidov et al., 2020; Lacerda et al., 2020; Yang et al., 2020; Xue et al., 2021).

We have previously established the Nanopore MinION as a tool for taxonomic metabarcoding of the plastisphere communities up to the species level (Davidov et al., 2020). In this continuation study we implemented a similar metabarcoding approach for the comprehensive high-resolution identification of marine prokaryotes and eukaryotes, including fungi. The current study had a modified experimental setup for testing the taxonomic composition of the biofilm on PE and PET versus glass reference, all with five replicates. This allowed us to identify organisms that were significantly more abundant on plastic.

## Materials and Methods

### Experimental Setup

**FIGURE 1:**
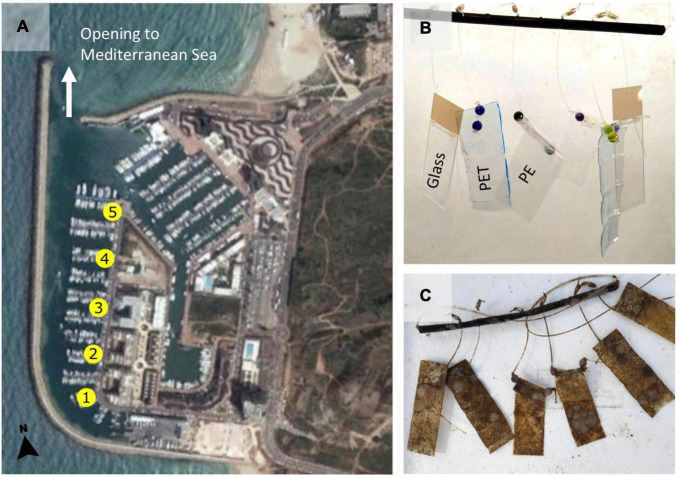
Experiment location and set up. **(A)** Locations of the five replicates that were placed from south to north in Herzliya Marina, Israel (32° 09′ 38.8″ N 34° 47′ 35.0″ E). **(B)** Each replicate consists of two 17 × 26 mm flat pieces of each of the three materials (PE,PET, and glass) tied with a fishing line to a straw and secured with plastic beads. This “mobile” of materials was secured to the dock such that the surfaces were submerged ∼0.5 m below the surface and 6–8 m above the marina bottom. **(C)** The materials after they were submerged for 30 days in Herzliya Marina water.

Two 17 × 26 mm flat pieces of each of the three materials were used, polyethylene (PE) from plastic food bags (Ziploc), polyethylene terephthalate (PET) from transparent drinking water bottles (Coca-Cola, 1.5 L) and glass microscope slides (Marienfeld). The surfaces were tied with a fishing line to a straw and secured with plastic beads to create the “mobile” structures. Five mobiles were then secured along the dock in Herzliya Marina, Israel (32° 09′ 38.8″ N 34° 47′ 35.0″ E) such that the surfaces were submerged ∼0.5 m below the water surface (Figure 1). After 1 month each material from the mobile was sampled for DNA extraction and metabarcoding or microscopy.

### Sample Collection and DNA Extraction

Each of the mobile materials were sampled in its entirety, gently washed three times for 5 min with filtered seawater to remove unbound material and separately processed in each of the subsequent assays and procedures described below. Seawater was sampled in proximity to each of the mobiles (five times) using sterile sampling bottles. 0.5 L of the sampled water was filtered on 0.22 μm polyethersulfone membrane (Millipore) using a 20 L/min pump (MRC). DNA was extracted using the phenol–chloroform extraction method (Debeljak et al., 2017; Supplementary Material, Protocol 1).

### PCR Amplification and Clean Up

One sample of each material (water filters, PE, glass and PET) from each mobile (five repeats) were subjected to PCR amplification with three sets of primers to amplify three barcode regions. The complete 16S rRNA gene was amplified using 27F and 1492R primers (Weisburg et al., 1991), with an expected product size of ∼1.5 kb. Primers 566F and 1289R (Hadziavdic et al., 2014) were used to amplify the V4 and V5 regions of the 18S rRNA gene, with an expected product size of ∼0.7 kb, and ITS86F and ITS4R (Op De Beeck et al., 2014) were used to amplify the fungal internal transcribed spacer 2 (ITS2) loci, with an expected product size of ∼0.4 kb. The amplification parameters and primer details are listed in Supplementary Material 1, Table 1. The reaction volume was 50 μL with 25–75 ng of template sample DNA. The PCR products were cleaned with QIAquick PCR Purification kit (QIAGEN) to meet the criteria of the MinION nanopore library preparation protocol (Karamitros and Magiorkinis, 2018).

### MinION Library Preparation and Multiplexed Nanopore Sequencing

The sequencing libraries were prepared using the Native barcoding amplicons protocol with EXP-NBD104 and SQK-LSK109 kits (Oxford Nanopore Technologies) according to Davidov et al. (2020). The 16S rRNA and 18S rRNA gene sequencing libraries were loaded to the same MinION flow cell in two batches: the first included the amplified 16S rRNA and 18S rRNA gene DNA barcodes of replicates 1, 3, and 5 (12 multiplexed libraries in total), and the second included the amplified 16S rRNA and 18S rRNA gene DNA barcodes of replicates 2 and 4 (8 multiplexed libraries in total). The two sequencing runs were separated by a washing step using EXP-WSH002 Kit (Oxford Nanopore Technologies). Each library was loaded onto to the MinION Nanopore Spot-on flow cell (FLO-MIN106D, version R9) and sequenced until reaching ∼7 Giga nucleotides (∼4 M reads). The ITS2 sequencing library was loaded to a new MinION flow cell and sequenced until reaching ∼1.3 Giga nucleotides (∼1 M reads). Base-calling for all libraries were done by the Guppy base calling software 3.3.3, using MinKNOW program with the “high accuracy” option. Raw reads were obtained in FAST5 and FASTq formats from which “pass” quality reads were subjected to further analysis.

### Sequence Analysis and Bioinformatics

Processing and analysis of reads was performed using MetONTIIME pipeline and QIIME2 plugins (Bolyen et al., 2019). The MetONTIIME pipeline was executed using the script “Launch_MinION_mobile_lab.sh” and “MetONTIIME.sh”. Configuration of MetONTIIME pipeline was performed using “config_MinION_mobile_lab” R script. Launch_MinION_mobile_lab.sh script was used for performing basecalling, demultiplexing, quality filtering, adapters and PCR primers trimming. This resulted in reads, filtered by length, quality and separated by barcodes into files in fastq format. Sequences were filtered based on read quality (min_quality; 16S and 18S, 9–11; ITS2, 8), and read length was restricted (amplicon_length X, lenfil_tol Y) based on read length histograms, to give the following range: 16S, 1399–1599 nucleotides; 18S, 605–755 nucleotides; ITS2, 100–1100 nucleotides. The following steps have been performed separately for each barcode. The MetONTIIME.sh script was run for performing dereplication, clustering and taxonomic classification. Sequences were clustered into consensus sequences with default MetONTIIME pipeline parameters (*de novo* strategy, clustering threshold parameter perc-identity 1). Consensus sequences of the 16S rRNA and 18S rRNA genes were assigned based on SILVA 132 database (Quast et al., 2013). The following parameters were used: taxonomic classifier Blast, max_accepts 1, query coverage 0.8 and identity threshold 0.85. Consensus sequences of the ITS barcode were classified against UNITE database V8 (Nilsson et al., 2019) using taxonomic classifier Blast, max_accepts 1, query coverage 0.8 and identity threshold 0.7. We chose max_accepts 1 for faster analysis, after testing 3 and 10 values for this command with raw data subsets which came up with the same results. Then, the feature tables of the used barcodes generated using the MetONTIIME pipeline were combined using the q2-feature-table plugin of the QIIME2, using the methods: merge, merge-seqs and merge-taxa. The code used is included in Supplementary Material 2. The featured tables were imported into R using phyloseq (R Core Team, 2018).

Sequences mapped to chloroplasts, mitochondria, eukaryotes, and “unknown organisms” were removed from the 16S rRNA gene analysis, while sequences mapped to bacteria (due to mitochondrial DNA) and “unknown organisms” were removed from the 18S rRNA gene and ITS analysis. Alpha diversity was estimated by richness [observed Operational Taxonomic Units (OTUs)], together with Shannon and Pielou diversity indexes using phyloseq (McMurdie and Holmes, 2013). After alpha diversity analysis, OTUs with a read count of 1 were excluded and the remaining OTUs were normalized to relative read abundance (dividing the number of reads for each sample by the total reads count). All further analyses were based on relative read abundance. For Beta diversity we used phyloseq (McMurdie and Holmes, 2013) to perform principle coordinates analysis (PCoA). This ordinated the sequences using the Bray–Curtis distance matrix to visualize multivariate structures of the communities. To test significant differences between the groups Permutational multivariate analysis of variance (PERMANOVA) tests were calculated using MicrobiomeAnalyst (Chong et al., 2020). Stacked bar plots and heatmaps were produced using MicrobiomeAnalyst (Chong et al., 2020) and Ampvis2 (Andersen et al., 2018), respectively. We used *limma* (Ritchie et al., 2015) to generate linear models and the *voom* function to transform normalized counts to log2-counts-per-million (logCPM) and estimate mean-variance of species between surface materials, similar to Calgaro et al. (2020). As the five “mobile” replicates were placed in slightly different locations within the marina, the mean location variance was removed from all measurements so the community differences due to surface type could be better seen. OTU differential abundance was visualized using Glimma (Su et al., 2017) and OTUs that had false discovery rate (FDR) adjusted *p*-value < 0.05 and log-fold-change of >0 were identified as differentially more abundant. Venn diagrams were created with InteractiVenn tool (Heberle et al., 2015), based on OTUs represented by at least two reads. All Nanopore MinION filtered reads analyzed in this project were deposited in the NCBI SRA database (Agarwala et al., 2016) under BioProject PRJNA680232 (accession numbers: SRX9553811–SRX9553850, SRX10393298–SRX10393305, and SRX11261845–SRX11261863).

### Biofilm Analysis and Microscopy

Scanning electron microscopy (SEM) was used to visualize the broad composition and the morphology of the biofilms on both PE and PET samples (Supplementary Material 1, Protocol 2). For fungal identification, Lactophenol Cotton Blue dye (Sigma-Aldrich) was applied to PE samples and visualized under a light microscope (Supplementary Material 1, Protocol 3). A modified crystal violet method (Supplementary Material 1, Protocol 4) was used for the indirect measurement of the relative biofilm biomass on the experimental samples.

## Results

### High Microbial Diversity Is Observed Within the 1-Month-Old Biofilm

**FIGURE 2:**
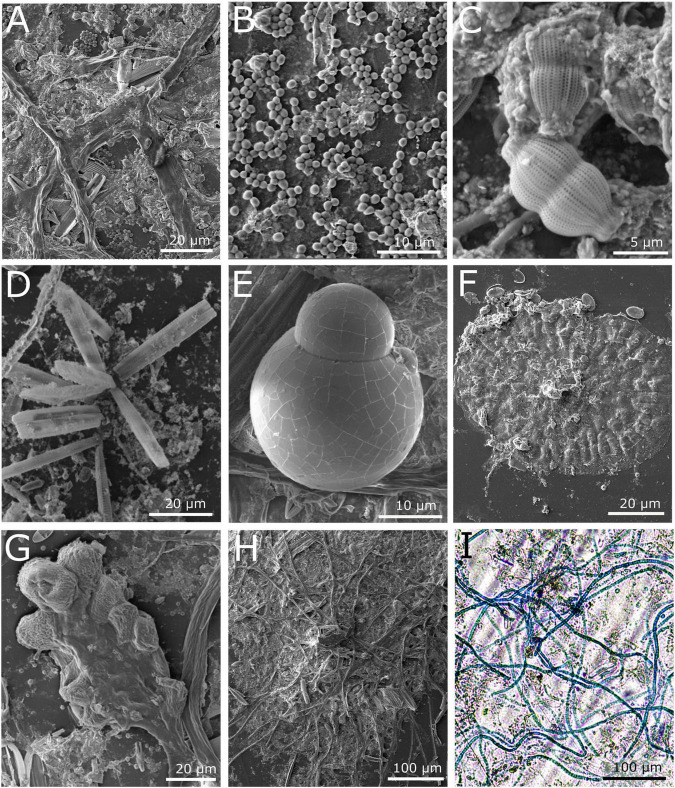
Microscopy images of the microbial community on PE or PET surfaces from marine waters after 1 month. **(A–H)** SEM images; **(A)** Biological coverage on PE surface after 1 month, **(B)** bacterial colonies on PET surface, **(C,D)** diatoms associated with biofilm on PE, **(C)** *Fragilariopsis sp.* **(D)** *Amphora sp.* **(E–H)** Images from PET; **(E)** a single cell eukaryote, **(F)** Bryozoan, **(G)** fruiting body, **(H)** multicellular filaments. **(I)** Light microscopy image of a network of fungal hyphae specifically stained with Lactophenol Cotton Blue.

Within 30 days from submersion in the marina waters, all substrates were heavily covered in biofilm (Figure 1C). The biomass was similar on all surfaces, as indicated by the crystal violet assay (Supplementary Material 1, Figure 1). SEM imaging revealed that the plastic surfaces were mostly covered with a likely bacterial biofilm (Figure 2A), probable exposed bacteria (Figure 2B) and multiple diatom species (Figures 2C–E). We were also able to identify single cell eukaryotes (Figure 2E), bryozoans (Figure 2F) and fungal-like fruiting bodies (Figure 2G). Furthermore, we observed multicellular filaments (Figure 2H), of which some were identified as algae based on the presence of chlorophyll and some were confirmed to be fungi hyphae by Lactophenol Cotton Blue staining (Figure 2I).

The nanopore sequencing run produced an average of 25,235 16S rRNA gene reads, 80,125 18S rRNA gene reads, and 24,777 ITS reads per sample. The average read length was 1,428 nucleotides for 16S rRNA gene, 669 for 18S rRNA gene and 369 for the ITS barcode. However, while both 16S rRNA and 18S rRNA gene amplicons had read length standard deviation of 24 and 20 accordingly, the ITS reads varied greatly in length with standard deviation of 154.5 nucleotides. The mapping rates of the ITS sequences to the reference databases were very low (3% on average) compared to the 16S rRNA and 18S rRNA gene mapping rates (both 99%), and the ITS sequences had a lower taxonomic resolution (Supplementary Material 1, Table 2).

Community complexity parameters including richness (number of observed OTUs), evenness (Pielou’s index) and diversity (Shannon’s index) were obtained for each of the sample types (water, PET, PE, and glass) based on 16S rRNA and 18S rRNA gene reads (Supplementary Material 1, Figure 2). For 16S rRNA gene sequence analyses, the water had significantly lower taxonomic evenness, contributing to lower OTU diversity (Shannon’s index) in comparison to the surface samples (Supplementary Material 1, Figure 2A). This agrees with previous environmental studies that showed the same trend (De Tender et al., 2015; Bryant et al., 2016; Didier et al., 2017). In contrary, the 18S rRNA gene analysis resulted in no significant difference among the samples in any of the diversity indexes (Supplementary Material 1, Figure 2B). Previous 18S rRNA gene based analyses report water communities as more diverse (Didier et al., 2017; Kettner et al., 2019; Dudek et al., 2020). Because of the low ITS mapping rates, we did not analyze the alpha diversity parameters for fungi.

**FIGURE 3:**
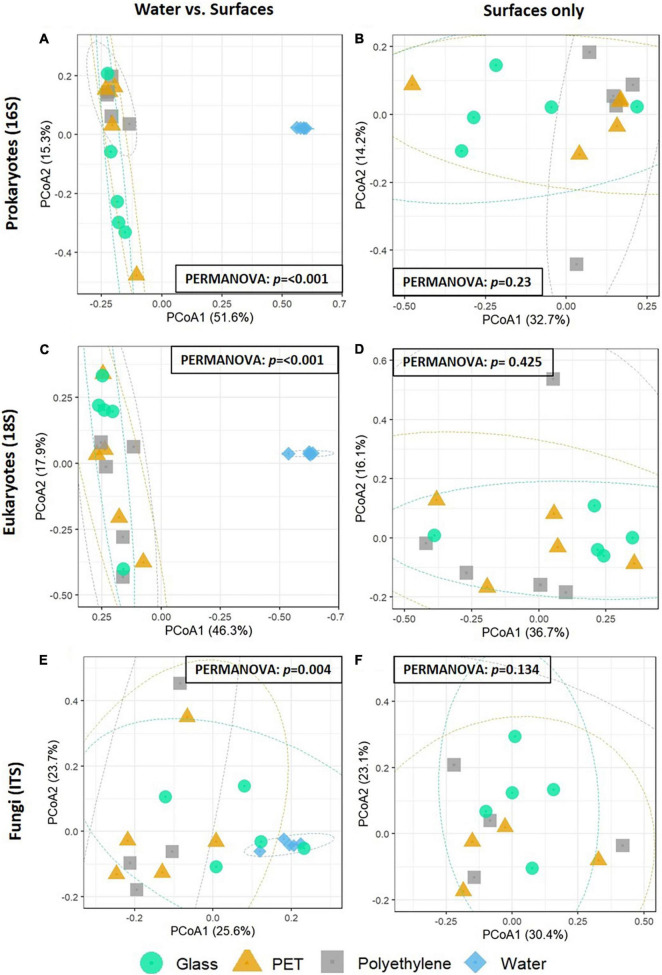
PCoA of the Bray-Curtis dissimilarities among the plastisphere communities of the samples (*N* = 5 per treatment). **(A,B)** 16S rRNA gene microbiomes with the water community (left) or without it (right). **(C,D)** 18S rRNA gene microbiomes with the water community (left) or without it (right). **(E,F)** ITS microbiomes with the water community (left) or without it (right). Results of PERMANOVA for significance between groups are shown on each plot.

To assess the similarities in the community composition between the samples, beta diversity analysis was performed using a PCoA analysis (Figure 3). In both 16S rRNA and 18S rRNA gene analyses, the five water samples formed distinctive clusters from the surface samples (Figures 3A–D). PERMANOVA analyses confirmed the water samples were significantly different from the other surfaces. In the ITS analysis, while the water samples showed homogeneity among themselves, they did not form a separate cluster from the other samples (Figures 3E,F). Despite this, PERMANOVA analyses confirmed the ITS water samples were significantly different from the other surfaces. When excluding the water samples, no distinct clusters were observed in any of the analyses (Figures 3B,D,F).

### Substrate Specificity of the 16S rRNA Gene Microbial Communities

**FIGURE 4:**
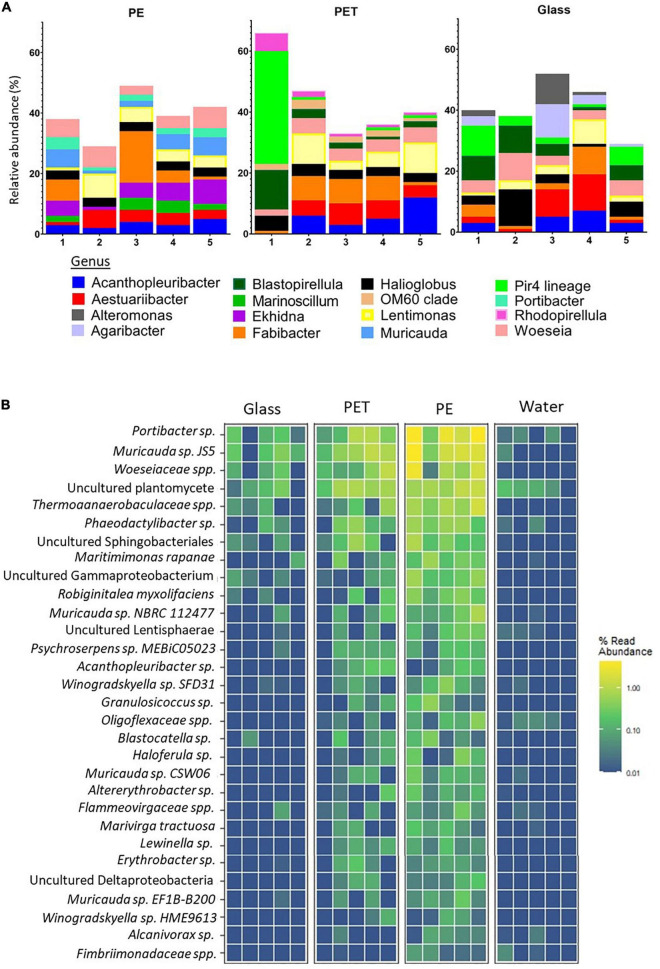
Substrate specificity of bacteria based on relative abundance of 16S rRNA gene mapped reads. **(A)** Relative abundance of top 10 abundant genera within each treatment. Produced using MicrobiomeAnalyst (Chong et al., 2020). **(B)** Heatmap of the bacteria with higher relative mapped read abundance in the PE samples Limma – *voom* was used to identify OTUs that had FDR adjusted *p*-value < 0.05 and log- fold- change of >0 on PET or PE compared to glass. The relative abundance of their mapped reads was then plotted for all samples and replicates.

The filtered, high quality 16S rRNA gene sequences were clustered into an average of 2,733 operational taxonomic units (OTUs) for PET, 21,129 for PE and 4,163 for glass, corresponding to 524, 754, and 677 classified organisms, respectively. The top 10 most abundant prokaryotic genera within each treatment, that were identified based on the relative abundance of mapped read counts, contain some different genera across the surfaces (Figure 4A). *Ekhidna*, *Muricauda*, and *Portibacter* were included in the top 10 most abundant genera on PE and not in the other surfaces, whereas *Rhodopirellula* and OM60 clade were included only in the PET top 10. Among the above genera, *Muricauda*, and *Rhodopirellula* have been previously reported as hydrocarbon-degrading bacteria (Jiménez et al., 2011; Didier et al., 2017; Laso-Pérez et al., 2019; de Araujo et al., 2021).

To identify species with preference to plastic, we searched for species with significantly higher read ratios in the PET and PE samples as opposed to glass. Linear discriminant analysis of our 16S rRNA gene sequences revealed 35 OTUs, corresponding to 30 bacteria database matches, that were significantly more abundant in the PE samples than in the glass samples (Figure 4B and Supplementary Material 1, Figure 3). Of the 30 bacteria matches, 9 were resolved at the species level and 10 at the genera level (leaving 11 of higher taxonomic classification).

Among the bacterial species that showed significantly higher read representation in the PE samples compared with the glass and water samples, *Muricauda sp. NBRC 112477* showed highest significance values (adjusted *p*-value = 0.003) (Supplementary Material 1, Table 3). We identified 3 other species of the genus *Muricauda*, which matches with the top 10 genera for PE (Figure 4A), and 2 species from the genus *Winogradskyella*, which has been previously reported as a hydrocarbon degrading genera (Wang et al., 2014). Other species that were significantly differentially abundant on PE included *Maritimimonas rapanae, Robiginitalea myxolifaciens* and *Psychroserpens sp. MEBiC05023*, all from the *Flavobacteriaceae* family, and *Marivirga tractuosa*. Of the unresolved sequences, *Alcanivorax sp.* is a well-known degrader of alkanes and petroleum (Yakimov et al., 2019). It was also recently shown that certain *Alcanivorax* species are able to grow and form biofilms when PE is the main carbon source (Delacuvellerie et al., 2019).

### Substrate Specificity of the 18S rRNA Gene Eukaryotic Communities

**FIGURE 5:**
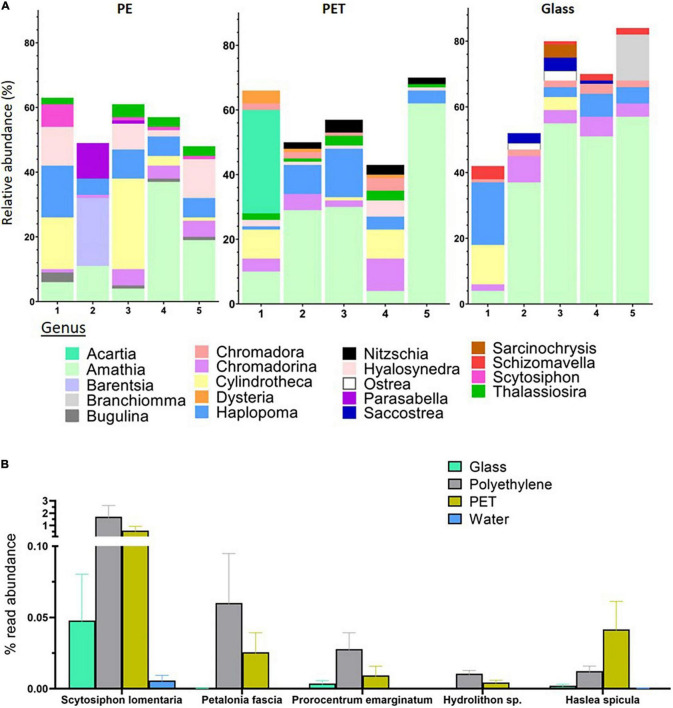
Substrate specificity of eukaryotes at the genus and species levels based on relative abundance of 18S rRNA gene mapped reads. **(A)** Relative abundance of top 10 abundant genera within each treatment. Produced using MicrobiomeAnalyst (Chong et al., 2020). **(B)** Eukaryotes with higher relative mapped read abundance in the plastic samples. Limma – *voom* was used to identify OTUs that had FDR adjusted *p*-value < 0.05 and log- fold- change of >0 on PET or PE compared to glass. The relative abundance of their mapped reads was then plotted for all samples.

The high quality 18S rRNA gene sequences were clustered into an average of 10,997 OTUs for PET, 11,608 for PE, and 7,956 for glass, corresponding to 730, 735, and 506 classified eukaryotes, respectively (Supplementary Table 2). Within the 18S rRNA gene mapped reads, the genera with the highest relative abundance across all surfaces was the bryozoan *Amathia.* However, there were also differences between the top 10 most abundant genera among the surfaces (Figure 5A). On PE, the most abundant genera included *Scytosiphon*, a genus of brown seaweed, the bryozoan *Bugulina*, the entoprocta *Barentsia* and the sabellid polychaete *Parasabella*. On the other hand, the top 10 genera on PET included the copepods genus *Acartia*, as well as the diatom genus *Nitzschia* and the ciliate genus *Dysteria.*

Linear discriminant analysis of our 18S rRNA gene sequences identified five OTUs, corresponding to four species and one genus, with significantly higher read ratios in the plastic samples compared to glass samples (Supplementary Material 1, Table 4). These species included two brown algae; *Petalonia fascia* and *Scytosiphon lomentaria*, with higher read ratios in both PET and PE samples. The benthic dinoflagellate *Prorocentrum emarginatum* and the red alga *Hydrolithon sp*. had higher relative representation in the PE samples, while reads mapped to the diatom *Haslea spicula* were found in relative higher ratios in the PET samples (Figure 5B).

### Marine Fungi Communities on Plastic Surfaces

For the identification of fungi we used the ITS2 barcode. The ITS2 locus has been shown to be the most suitable taxonomic barcode for the characterization of fungal communities and has been previously used for the identification of fungi from marine plastic debris (Op De Beeck et al., 2014; Davidov et al., 2020). Due to the limitations of the available databases for marine fungi, only 3% of the reads were mapped to reference sequences. Despite this, our analysis still identified an average of 57 fungal OTUs in the PET samples and 58 in the PE samples, of which 23 and 25, respectively, were classified to the species level. We also identified an average of 87 fungal OTUs in the glass samples and 114 in the water samples, of which 29 and 41, respectively, were classified to the species level (Supplementary Material 1, Table 2).

**FIGURE 6:**
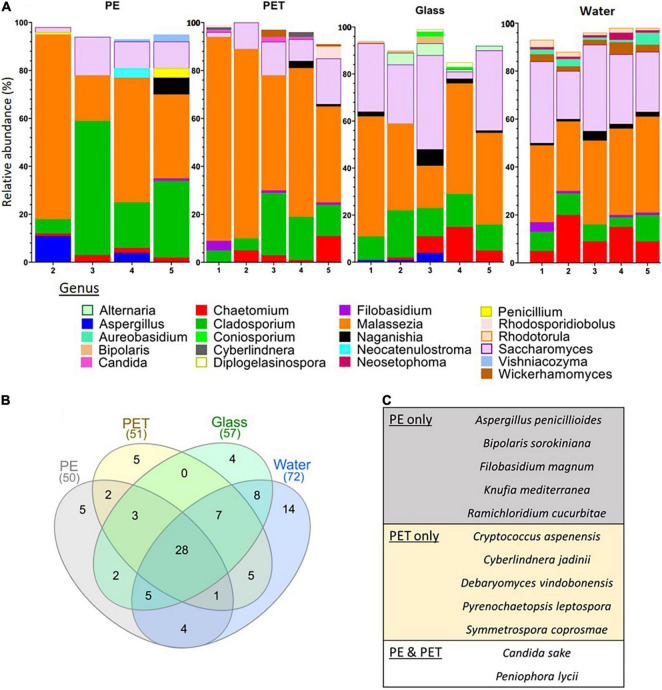
ITS fungal metabarcoding analysis. **(A)** Top 10 abundant fungi genera based on ITS metabarcoding. **(B)** Shared and unique fungal OTUs among the surfaces and the water. **(C)** Fungi corresponding to the OTUs present only on PE, PET or PE, and PET.

Similar to the 16S rRNA and 18S rRNA gene based beta-diversity analyses, the fungi (barcoded with ITS) analysis did show distinction between the taxonomic composition of the water vs. the composition of the biota associated with surfaces (Figure 3E). Furthermore, within the ITS mapped reads, there were differences between the top 10 relatively abundant genera across the surfaces (Figure 6A). On PE, the most abundant genera included *Neocatenulostroma*, *Penicillium*, and *Vishniacozyma.* Whereas in the top 10 genera of the PET samples we identified *Candida*, *Cyberlindnera*, and *Rhodosporidiobolus.* Due to the limited mapping results, differential abundance analysis was not performed. Nevertheless, of all 316 OTUs identified, five were found only on PE (Figure 6B) corresponding to species *Aspergillus penicillioides, Bipolaris sorokiniana, Filobasidium magnum, Knufia mediterranea* and *Ramichloridium cucurbitae* (Figure 6C). Another five OTUs were only found on the PET samples corresponding to *Cryptococcus aspenensis, Cyberlindnera jadinii, Debaryomyces vindobonensis, Pyrenochaetopsis leptospora*, and *Symmetrospora coprosmae*. Another two OTUs corresponding to the species *Candida sake*, and *Peniophora lycii* were identified in the PET and the PE samples but not in the glass samples and the water.

## Discussion

In this study, we analyzed 1-month-old Mediterranean Sea plastisphere communities and screened for prokaryotic and eukaryotic species, including fungi, with differential abundance on PET and PE plastic surfaces compared to glass. Many studies have focused on the search for plastic-specific organisms [reviewed in Latva et al. (2021)]. However, very few resulted in the identification of the taxonomic levels of species. For achieving a higher taxonomic resolution, including the species level, we used longer barcode regions that were sequenced with the nanopore MinION platform. Additionally, we characterized the composition of the fungal biome using a shorter, well-established barcode within the ITS2 locus.

Previous 16S rRNA gene, 18S rRNA gene and ITS metabarcoding analyses have repeatedly demonstrated that the plastisphere is a separate ecological niche from the surrounding water (Zettler et al., 2013; Kettner et al., 2017; Dussud et al., 2018; Frère et al., 2018). Our metabarcoding beta diversity analysis supports this observation. We would like to note that a definitive conclusion cannot be drawn for the fungal communities because of the low mapping rates of the ITS sequences (∼3%). Other studies that have used the ITS barcode resulted in similarly low mapping rates of 0.01–4% (Kettner et al., 2017; Lacerda et al., 2020; Wang et al., 2021) which is attributed to the limited taxonomic coverage of the available fungal databases, especially when it comes to marine fungi (Hawksworth and Lücking, 2017).

So far, most environmental studies did not find conclusive differences between the microbial communities on plastic vs. glass surfaces (Oberbeckmann et al., 2016; Pinto et al., 2019; Erni-Cassola et al., 2020; Laverty et al., 2020; Zhao et al., 2021). On the other hand, studies in enclosed or semi-enclosed systems often showed differential taxonomic representation (Kirstein et al., 2018, 2019; Ogonowski et al., 2018; Pinto et al., 2020). These differences between the two types of studies may be due to the milder masking effects of environmental factors under controlled lab conditions. For this reason, we find that the location of our experiment, within the semi-protected environment of the marina that is connected to the open sea, may serve as an ideal location for such comparative studies of the natural environment.

To identify the high-resolution differences in the taxonomic composition of the microbiomes, we first analyzed the top 10 most abundant genera in each surface. This analysis resulted in several genera that were recurrently identified on one substrate and not in the other. However, it has been established that the OTUs contributing to the most dissimilarity between substrates are not necessarily the most abundant ones (e.g., Kirstein et al., 2018, 2019). Therefore, it is important to use the appropriate statistical tools to identify differentially abundant species. Here we took advantage of the *limma* package, which implements statistical algorithms developed for the analysis of differential expressed genes. These specialized algorithms make statistical conclusions more reliable when the number of samples are small and have different levels of variability and complex set ups (Ritchie et al., 2015). Our analysis identified 30 prokaryotes for which the relative abundance of 16S rRNA gene reads were significantly differentially abundant in the PE samples compared to the glass samples. Many of which belong to genera that have been previously reported in association with plastic communities including, *Maritimimonas* (De Tender et al., 2017), *Saprospiraceae*, *Flammeovirgaceae*, and *Lewinella* [reviewed in Roager and Sonnenschein (2019)] as well as *Fulvivirga* (Tourova et al., 2020) and *Cyclobacteriaceae* (Miao et al., 2019). Moreover, our differential abundance analysis identified bacteria that have been suggested to be hydrocarbon and plastic-degrading including genera *Muricauda* (Didier et al., 2017), *Winogradskyella* (Wang et al., 2014) and *Alcanivorax* (Delacuvellerie et al., 2019). Lastly, there were 16 organisms that were not reported previously as plastic-associated, of which 5 were uncultured. In a comparison among the plastic surfaces vs. glass, differentially abundant bacteria were identified only in the PE samples but not on PET. We hypothesize that because PE floats whereas both PET and glass usually sink, it is exposed to different environmental conditions, such as direct sunlight, and therefore it has been selected for colonization by a slightly different set of microorganisms.

The same analysis for the 18S rRNA gene sequences identified five eukaryotes that were differentially abundant on PE and PET compared with glass including two species of brown algae, *Scytosiphon lomentaria* and *Petalonia fascia* which were previously identified in the plastic microbiome (Ibabe et al., 2020). The dinoflagellate *P. emarginatum* and the red algae *Hydrolithon sp*., that had significantly different read ratios on PE, have also been reported to dominate plastisphere communities (Dudek et al., 2020). The diatom *Haslea spicula*, which was significantly differentially abundant on the PET surfaces is a mobile pennate diatom that is known to colonize artificial surfaces (Winfield et al., 2018). The presence of diatoms on marine plastic has been repeatedly shown (e.g., Oberbeckmann et al., 2014; Reisser et al., 2014; Davidov et al., 2020; Dudek et al., 2020) and was clearly observed in our SEM and light microscopy imaging.

The fungal biome on marine plastic has been so far understudied and only a handful have analyzed the ITS genes (e.g., Zhang et al., 2015; De Tender et al., 2017; Davidov et al., 2020; Lacerda et al., 2020). Our ITS metabarcoding analyses and lactophenol cotton blue staining showed the plastic surfaces in the marina were colonized by a highly developed network of fungi, mostly of the genera *Malassezia* (phylum Basidiomycota), *Cladosporium* and *Saccharomyces* (phylum Ascomycota). The presence of *Cladosporium* on plastic debris was previously shown (De Tender et al., 2017; Lacerda et al., 2020; Xue et al., 2021). 12 fungal OTUs were found only on the plastic samples including the species *Pyrenochaetopsis leptospora*, *Candida sake*, *Debaryomyces vindobonensis, Aspergillus penicillioides*, and *Bipolaris sorokiniana*, a wheat pathogen that causes leaf spot disease (Ye et al., 2019). *Peniophora lycii*, a species that was found on both PE and PET, but not on glass or water, has recently been shown to secrete three laccase isozymes (Glazunova et al., 2020), that may be capable of breaking down non-hydrolyzable plastics such as PE (Inderthal et al., 2021). Many marine fungi can degrade complex hydrocarbons [reviewed in Amend et al. (2019)] and often dominate in oil polluted environments (Bik et al., 2012; McGenity et al., 2012). So far only one marine fungi, *Zalerion maritimum* has been suggested to degrade plastic (PE) in laboratory conditions (Paço et al., 2017; Santacruz-Juárez et al., 2021). Although molecular mechanisms still remain to be identified. Fungi are an abundant and active component of the ocean environment with plastic degradation potential that warrants further investigation.

While DNA metabarcoding of the plastisphere biome continues to be a favorable approach for its taxonomic composition characterization it needs to be fine-tuned to be effective in the identification of plastic-specific genera and species. Refining the resolution and the scope of this approach will provide useful information that can be the basis for species-targeted studies to unveil the molecular mechanism for plastic colonization and metabolism.

## Data Availability Statement

The datasets presented in this study can be found in online repositories. The names of the repository/repositories and accession number(s) can be found in the article.

## Author Contributions

KD, EI-K, and MO set up the experiment. KM, KD, EI-K, and SI did the bench work. KM wrote the main manuscript. YK and KM did the bioinformatic analyses. MO and MS-D reviewed and edited the manuscript, and received the fundings. All authors have given approval to the final version of the manuscript.

## Conflict of Interest

The authors declare that the research was conducted in the absence of any commercial or financial relationships that could be construed as a potential conflict of interest.

## Publisher’s Note

All claims expressed in this article are solely those of the authors and do not necessarily represent those of their affiliated organizations, or those of the publisher, the editors and the reviewers. Any product that may be evaluated in this article, or claim that may be made by its manufacturer, is not guaranteed or endorsed by the publisher.
