# Supplementary material for: High-Resolution Screening for Marine Prokaryotes and Eukaryotes With Selective Preference for Polyethylene and Polyethylene Terephthalate Surfaces
Source: Front Microbiol. 2022 Apr 12;13:845144. doi: 10.3389/fmicb.2022.845144 (PMC9042255; doi:10.3389/fmicb.2022.845144)
Supplement: Supplementary file 2 [file Data_Sheet_2.docx]

Supplementary material 2

**The code used for sequence analysis and bioinformatics**

# 16S

/opt/software/MetONTIIME/Launch_MinION_mobile_lab.sh /home/User/MinION_data/16S_mobile_1_3_5/fast5_pass

/opt/software/MetONTIIME/Launch_MinION_mobile_lab.sh /home/User/MinION_data/16S_mobile_2_4/fast5_pass

nohup /opt/software/MetONTIIME/MetONTIIME.sh /home/User/MetONTIIME/16S_mobile /home/User/MetONTIIME/sample-metadata.tsv /home/User/MetONTIIME/DB/Silva132/16S/silva_132_99_16S_sequence.qza /home/User/MetONTIIME/DB/Silva132/16S/silva_132_99_16S_taxonomy.qza 30 Blast 1 0.8 0.85 &

# export:

qiime tools export \

--input-path /home/User/MetONTIIME/16S_mobile/table.qza \

--output-path /home/User/MetONTIIME/16S_mobile/export

biom convert -i /home/User/MetONTIIME/16S_mobile/export/feature-table.biom -o /home/User/MetONTIIME/16S_mobile/export/otu_table.txt --to-tsv

qiime tools export \

--input-path /home/User/MetONTIIME/16S_mobile/taxonomy.qza \

--output-path /home/User/MetONTIIME/16S_mobile/export

# 18S

/opt/software/MetONTIIME/Launch_MinION_mobile_lab.sh /home/User/MinION_data/18S_mobile_1_3_5/fast5_pass

/opt/software/MetONTIIME/Launch_MinION_mobile_lab.sh /home/User/MinION_data/18s_mobile_2_4/fast5_pass

nohup /opt/software/MetONTIIME/MetONTIIME.sh /home/User/MetONTIIME/18S_mobile /home/User/MetONTIIME/sample-metadata.tsv /home/User/MetONTIIME/DB/Silva132/18S/silva_132_99_18S_sequence.qza /home/User/MetONTIIME/DB/Silva132/18S/silva_132_99_18S_taxonomy.qza 30 Blast 1 0.8 0.85 &

# export:

qiime tools export \

--input-path /home/User/MetONTIIME/18S_mobile/table.qza \

--output-path /home/User/MetONTIIME/18S_mobile/export

biom convert -i /home/User/MetONTIIME/18S_mobile/export/feature-table.biom -o /home/User/MetONTIIME/18S_mobile/export/otu_table.txt --to-tsv

qiime tools export \

--input-path /home/User/MetONTIIME/18S_mobile/taxonomy.qza \

--output-path /home/User/MetONTIIME/18S_mobile/export

# ITS

/opt/software/MetONTIIME/Launch_MinION_mobile_lab.sh /home/User/MetONTIIME/ITS_mobile_BC01-12/fast5_pass

/opt/software/MetONTIIME/Launch_MinION_mobile_lab.sh /home/User/MetONTIIME/ITS_mobile_BC13-19/fast5_pass

nohup /opt/software/MetONTIIME/MetONTIIME.sh /home/User/MetONTIIME/ITS_mobile /home/User/MetONTIIME/sample-metadata.tsv /home/User/UNITE/unite-ver8-dynamic-sequences-04.02.2020-dev.qza /home/User/UNITE/unite-ver8-dynamic-taxonomy-04.02.2020-dev.qza 30 Blast 1 0.7 0.7 &

# export:

qiime tools export \

--input-path /home/User/MetONTIIME/ITS_mobile/table.qza \

--output-path /home/User/MetONTIIME/ITS_mobile/export

biom convert -i /home/User/MetONTIIME/ITS_mobile/export/feature-table.biom -o /home/User/MetONTIIME/ITS_mobile/export/otu_table.txt --to-tsv

qiime tools export \

--input-path /home/User/MetONTIIME/ITS_mobile/taxonomy.qza \

--output-path /home/User/MetONTIIME/ITS_mobile/export

# import:

library("phyloseq")

otu <- read.table(file = "otu_table.txt", header = TRUE)

tax <- read.table(file = "taxonomy.tsv", sep = '\t', header = TRUE)

combined_table <- merge(otu, tax, by.x = c("OTUID"), by.y = c("OTUID"))

write.table(combined_table, file = "combined_table", sep = '\t', col.names = TRUE, row.names = FALSE)

# Split combined_table.txt file into two files: otu_matrix.csv, taxonomy.csv

otu_table <- read.csv("otu_matrix.csv", sep=",", row.names=1)

otu_table <- as.matrix(otu_table)

taxonomy = read.csv("taxonomy.csv", sep=",", row.names=1)

taxonomy<-as.matrix(taxonomy)

metadata=read.csv("metadata.csv",sep=",",row.names=1)

OTU = otu_table(otu_table, taxa_are_rows = TRUE)

TAX = tax_table(taxonomy)

META = sample_data(metadata)

physeq = phyloseq(OTU,TAX,META)
